# Effect of estrogen receptor β agonists on proliferation and gene expression of ovarian cancer cells

**DOI:** 10.1186/s12885-017-3246-0

**Published:** 2017-05-08

**Authors:** Susanne Schüler-Toprak, Christoph Moehle, Maciej Skrzypczak, Olaf Ortmann, Oliver Treeck

**Affiliations:** 1 Department of Obstetrics and Gynecology, University Medical Center Regensburg, Landshuter Str. 65, 93053 Regensburg, Germany; 2 Center of Excellence for Fluorescent Bioanalytics (KFB), Am BioPark 9, 93053 Regensburg, Germany; 3 Second Department of Gynecology, Medical University of Lublin, Jaczewskiego 8, 20-090 Lublin, PL Poland

**Keywords:** Estrogen receptor beta, Ovarian cancer, Estrogen receptor beta agonists

## Abstract

**Background:**

Estrogen receptor (ER) β has been suggested to affect ovarian carcinogenesis. We examined the effects of four ERβ agonists on proliferation and gene expression of two ovarian cancer cell lines.

**Methods:**

OVCAR-3 and OAW-42 ovarian cancer cells were treated with the ERβ agonists ERB-041, WAY200070, Liquiritigenin and 3β-Adiol and cell growth was measured by means of the Cell Titer Blue Assay (Promega). ERβ expression was knocked down by transfection with specific siRNA. Additionally, transcriptome analyses were performed by means of Affymetrix GeneChip arrays. To confirm the results of DNA microarray analysis, Western blot experiments were performed.

**Results:**

All ERβ agonists tested significantly decreased proliferation of OVCAR-3 and OAW-42 cells at a concentration of 10 nM. Maximum antiproliferative effects were induced by flavonoid Liquiritigenin, which inhibited growth of OVCAR-3 cells by 31.2% after 5 days of treatment, and ERB-041 suppressing proliferation of the same cell line by 29.1%. In OAW-42 cells, maximum effects were observed after treatment with the ERβ agonist WAY200070, inhibiting cell growth by 26.8%, whereas ERB-041 decreased proliferation by 24.4%. In turn, knockdown of ERβ with specific siRNA increased cell growth of OAW-42 cells about 1.9-fold. Transcriptome analyses revealed a set of genes regulated by ERβ agonists including ND6, LCN1 and PTCH2, providing possible molecular mechanisms underlying the observed antiproliferative effects.

**Conclusion:**

In conclusion, the observed growth-inhibitory effects of all ERβ agonists on ovarian cancer cell lines in vitro encourage further studies to test their possible use in the clinical setting.

## Background

Ovarian cancer is the fifth most common cause of death because of cancer in women and is the leading cause of death from gynaecological malignancy in the developed world [1]. Due to missing screening methods and its aggressive behaviour, a vast number is diagnosed at an advanced stage [2]. Steroid hormones have an influence on ovarian cancer cells [3] and it has been shown that 40–60% of ovarian cancers express estrogen receptor (ER) α [4, 5]. In advanced stages the selective estrogen receptor modulator tamoxifen is used in patients as a well-tolerated and also effective treatment [6–8]. Moreover, use of peri- and postmenopausal hormone therapy has been shown to increase ovarian cancer risk [9]. One extra ovarian cancer case per 1000 users can be observed in women who use hormone therapy for 5 years after the age of 50 years [9].

Investigating the underlying mechanisms, it is inevitable to consider the two ER types, ERα and β. So far, little is known about the molecular mechanisms of ERβ function in ovaries and ovarian cancers. However, it has been shown that both receptor types exert different biological functions [10, 11]. Given that ERβ is able to counteract ERα signaling in some settings, loss of ERβ is thought to enhance ERα-mediated proliferation of hormone-dependent cancer cells [12]. Moreover, the influence of ERb signaling on apoptosis pathways has been shown [13].

Comparing normal ovarian tissue with epithelial ovarian cancers, a loss of ERβ expression and a decrease in ERβ/ERα ratio can be observed [14–16]. Furthermore, in metastases of ovarian cancers a complete loss of ERβ was observed, whereas in the corresponding primary tumors low expression levels were still measurable [15]. A positive correlation of ERβ expression with survival has been shown in ovarian cancer patients as well as animal models [17, 18].

In vitro studies on other hormone-dependent tumors as breast and prostate cancers revealed a tumor suppressive role of ERβ [10, 19]. Fewer reports suggest that this receptor plays a similar role in ovarian cancer. Recently, we investigated the effect of ERβ overexpression on the SK-OV-3 ovarian cancer cells. Particularly overexpression of ERβ1 inhibited growth and motility of these cells and induced apoptosis. In addition, we observed specific changes in gene expression. Interestingly, the antitumoral effects of ERβ were independent of estradiol and functional ERα. However, we were able to show an increased transcription of cyclin-dependent kinase inhibitor 1, a decrease in cyclin A2 transcripts and an up-regulation of fibulin 1c [20].

In another study, proliferation of ERα expressing BG − 1 ovarian cancer cells decreased after reintroduction of ERβ expression [17]. An increased expression of ERβ was associated with a decreased number of cells in S phase, whereas more cells were found in the G2/M phase. Also the cell cycle regulators cyclin D1 and A2 were affected by ERβ expression. When ERβ was reintroduced, total retinoblastoma (Rb), phosphorylated Rb and phospho-AKT content decreased. A part of the antiproliferative effect of ERβ was explained by the strong inhibition of ERα activity and expression by ERβ [17, 21]. To examine the role of ERβ in a more physiological model of ovarian carcinogenesis, Bossard et al. orthotopically transplanted ERβ expressing ovarian cancer cells in ovaries of Nude mice, which reduced both tumor growth and the presence of tumor cells in sites of metastasis, and led to improved survival [17].

The suggested role of ERβ as tumor suppressor and the observed decrease of expression in ovarian cancer cells raise the question, whether ERβ expression in these cells might be high enough to make this receptor a potential target in ovarian cancer therapy. Thus, we investigated the effect of ERβ agonists on proliferation and gene expression of two ovarian cancer cell lines.

## Methods

### Material

The human ovarian cancer cell line OVCAR-3 was obtained from American Type Culture Collection (ATCC #HTB-161, Manassas, USA), and OAW-42 ovarian cancer cells were obtained from Sigma Aldrich (#85073102, St. Louis, USA). The cells were maintained in phenol red-free DMEM culture medium that was obtained from Invitrogen (Karlsruhe, Germany) containing FCS that was purchased from PAA (Pasching, Austria). RNeasy Mini Kit was obtained from Qiagen (Hilden, Germany). Transfectin reagent was obtained from BioRad (Hercules, USA). OptiMEM medium were purchased at Invitrogen (Karlsruhe, Germany). ESR2 and control siRNAs were from Ambion (Life Technologies, USA). Serum Replacement 2 (SR2) cell culture supplement and 17-β estradiol were from Sigma-Aldrich (Deisenhofen, Germany). ERβ agonists ERB-041 and WAY-200070 were from Tocris (Bristol, UK). 5α-androstane-3β, 17β-diol (3β-Adiol) was from Sigma (Deisenhofen, Germany) and Liquiritigenin from Extrasynthese (Lyon, France).

### Cell culture, transfection and proliferation assays

OVCAR-3 and OAW-42 cells were maintained in DMEM/F12 medium supplemented with 10% FCS at 37 °C in a humidified atmosphere containing 5% CO_2_. For transfection, 4 × 10^5^ cells per well of a 6-well dish were seeded in DMEM/F12 containing 10% FCS. The next day, 2 ml fresh culture medium was added to the cells. 5 μl Transfectin reagent (BioRad) and a mix of three ESR2 siRNAs (10 nM each) were used to prepare transfection solution in OptiMEM medium (Invitrogen). The siRNA mix contained three different ESR2-specific Silencer siRNAs (siRNA IDs 145,909, 145,910, 145,911, Ambion), targeting exons 1, 2 and 3 of ESR2 mRNA. As a negative control, Silencer Negative control siRNA #1 (Ambion) was used. Gene knockdown of ESR2 was verified by means of Western blot analysis 72 h after siRNA treatment as described below. For cell proliferation assays, cells cultured in DMEM/F12 supplemented with 10% FBS or serum replacement 2, both containing 0.1 nM E2, were seeded in 96-well plates in triplicates (1000 cell/well). For agonist analyses, ERβ agonists were added in a 10 nM concentration 1day later. The relative numbers of viable cells were measured on days 0, 3, 4, 5, 6 and 7 using the fluorimetric, resazurin-based Cell Titer Blue assay (Promega) according to the manufacturer’s instructions at 560Ex/590Em nm in a Victor3 multilabel counter (PerkinElmer, Germany). Cell growth was expressed as percentage of cells transfected with negative control siRNA. Growth data were statistically analyzed by the Kruskal–Wallis one-way analysis of variance.

### Antibodies and Western blot analysis

OAW-42 and OVCAR-3 cells were lysed in RIPA buffer (1% (*v*/v) Igepal CA-630, 0.5% (*w*/*v*) sodium deoxycholate, 0.1% (*w*/*v*) sodium dodecyl sulphate (SDS) in phosphate-buffered solution (PBS) containing aprotinin and sodium orthovanadate. Aliquots containing 10 μg of protein were resolved by 10% (*w*/*v*) SDS–polyacrylamide gel electrophoresis, followed by electrotransfer to a PVDF hybond (Amersham, UK) membrane. Immunodetection was carried out using monoclonal ERβ (ESR2) antibody 14C8 (ab288, Abcam, Germany), diluted 1:100 in PBS containing 5% skim milk (*w*/*v*), ERα (ESR1) antibody 6F11 (ab9269, Abcam, Germany) (1:500), lipocalin-1 (LCN1) antibody STJ96584 by St John’s Laboratory (London, UK) (1:300), Patched 2 (PTCH2) antibody ABIN1673339 (1: 500) by antibodies-online (Aachen, Germany), Mitochondrially Encoded NADH Dehydrogenase 6 (MT-ND6) antibody ABIN311275 (1:1000) by antibodies-online (Aachen, Germany), β-actin (ACTB) antibody (clone AC-74) from Sigma Aldrich (Munich, Germany) followed by horseradish peroxidase conjugated secondary antibody (1:50,000) which was detected using chemiluminescence (ECL) system (Amersham, Buckinghamshire, UK). The Western blot results from three independent protein isolations were densitometrically analyzed using ImageJ [22] and expressed in percentage of cell treated with a vehicle control.

### GeneChip™ microarray assay

Processing of the RNA samples (two biological replicates from OVCAR-3 and OAW-42 cells treated with E2 (0.1 nM) in combination with ERβ agonists (10 nM) or vehicle controls for 48 h) was performed at the local Affymetrix Service Provider and Genomics Core Facility, “KFB - Centre of Excellence for Fluorescent Bioanalytics” (Regensburg, Germany; www.kfb-regensburg.de).

Samples were prepared for microarray hybridization as described in the Affymetrix GeneChip^®^ Whole Transcript (WT) Sense Target Labelling Assay manual. Double-stranded cDNA was generated from 300 ng of total RNA. Subsequently, cRNA was synthesized using the WT cDNA Synthesis and Amplification Kit (Affymetrix). cRNA was purified and reverse transcribed into single-stranded (ss) DNA. Subsequently a combination of uracil DNA glycosylase (UDG) and apurinic/apyrimidinic endonuclease 1 (APE 1) was used to fragment ssDNA, which was afterwards labelled with biotin (WT Terminal Labelling Kit, Affymetrix). In a rotating chamber, 2.3 μg DNA were hybridized to the GeneChip Human Gene 1.0 ST Array (Affymetrix) for 16 h at 45 °C. After washing and staining the hybridized arrays in an Affymetrix Washing Station FS450 using preformulated solutions (Hyb, Wash & Stain Kit, Affymetrix), the fluorescent signals were measured with an Affymetrix GeneChip^®^ Scanner 3000-7G.

### Microarray data analysis

Summarized probe signals were created by using the RMA algorithm in the Affymetrix GeneChip Expression Console Software and exported into Microsoft Excel. Data was then analysed using Ingenuity IPA Software (Ingenuity Systems, Stanford, USA) and Genomatix Pathway Analysis software (Genomatix, Munich, Germany). Genes with more than 2-fold changed mRNA levels after ERβ knockdown in both biological replicates were considered to be differentially expressed and were included in the analyses.

## Results

### Expression of ERα and β in OVCAR-3 and OAW-42 cells

**Fig. 1:**
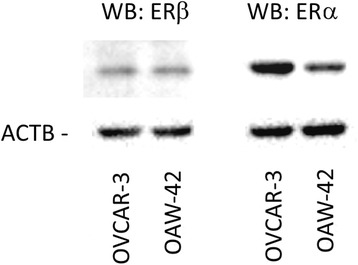
Expression of ERβ and ERα in OVCAR-3 and OAW-42 ovarian cancer cells. Expression of the indicated receptors was examined by means of Western blot analysis. Levels of β-Actin (AKTB) were determined as internal control. Aliquots containing 10 μg of protein isolated from both cell lines were resolved by 10% (*w*/*v*) SDS–polyacrylamide gel electrophoresis, followed by electrotransfer to a PVDF hybond membrane (Amersham, UK)

First, we tested expression of ERα and ERβ in the employed ovarian cancer cell lines OVCAR-3 and OAW-42. Western blot experiments demonstrated that both cell lines expressed ERβ protein at similar levels, whereas ERα protein levels were about 4-fold higher in OVCAR-3 cells (Fig. 1).

### ERβ agonists decreased proliferation of OVCAR-3 and OAW-42 cells

OVCAR3 and OAW-42 cells were treated with four different ERβ agonists, ERB-041, WAY-200070, Liquiritigenin and 3β-Adiol. Culture medium contained either 10% FCS or defined growth factor-free serum replacement, both containing E2 (0.1 nM). After treatment of OVCAR-3 and OAW-42 cells with the ERβ agonists, all of these drugs were observed to significantly decrease proliferation in both cell lines at a concentration of 10 nM. We decided to test this concentration only, because the EC50 values for ERβ binding of all drugs are in the low nanomolar range, and we wanted to rule out activation of ERα by higher drug concentrations, which could be able to increase proliferation.

**Fig. 2:**
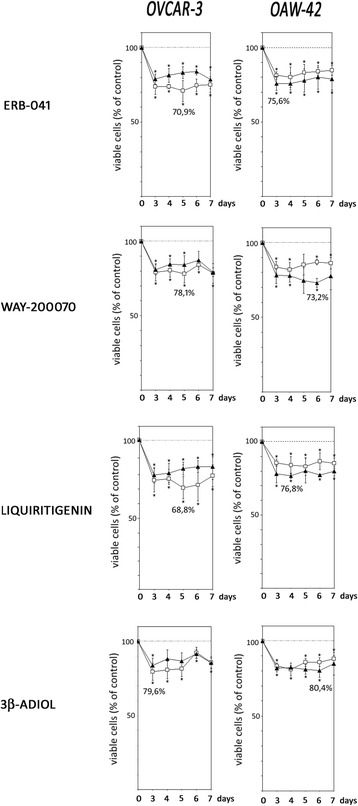
Effects of ERβ-agonists on growth of OVCAR-3 and OAW-42 ovarian cancer cells. OVCAR-3 and OAW-42 cells cultured in medium containing 10% FCS (open squares) or defined serum replacement SR2 (filled triangles) were treated with 10 nM of ERB-041, WAY-200070, Liquiritigenin or 3β-Adiol as indicated for up to 7 days and relative numbers of viable cells were determined by means of the fluorimetric CellTiter-Blue^®^ Assay (Promega). Data are expressed in percent of the vehicle controls (*n* = 4; * *P* < 0.05 vs. control; ** *P* < 0.01 vs. control)

In OVCAR-3 cells, maximum growth-inhibitory effects were induced by Liquiritigenin, which decreased the number of viable cells down to 68.8% after 5 days of treatment in medium supplemented with 10% FCS, when compared to cells treated with vehicle (Fig. 2). In SR2 containing medium, Liquiritigenin reduced viable cell numbers down to 78.6% on day 7. Treatment of OVCAR-3 cells with ERB-041 decreased the number of viable cells to 70.9% (day 5) in FCS containing medium and down to 78.6% (day 7) when cultured with defined serum replacement. WAY200070 treatment of OVCAR-3 cells inhibited proliferation to 78.1% on day 5 in FCS containing medium (79.3% on day 7 in SR2 containing medium). When 3β-Adiol was added, maximum effects were observed on day 3 with a decrease of viable cells down to 79.6% or 83.8% in FCS or SR2 containing medium, respectively.

All ERβ agonists tested also exerted significant growth inhibitory effects on OAW-42 cells. In contrast to OVCAR-3 cells, these effects were more pronounced in defined serum-free medium (Fig. 2). Maximum antiproliferative effects were observed in OAW-42 cells treated with WAY200070 on day 6, with a decrease of viable cell numbers to 73.2% in SR2 containing medium (81.8% on day 4 in FCS containing medium). Treatment with ERB-041 led to a maximum reduction of viable cells on day 3 down to 75.6% in SR2 and 81.3% in FCS containing medium. When OAW-42 cells were treated with Liquiritigenin, we observed a reduction of viable cell numbers down to 76.8% on day 4 (in FCS; 83.1% in SR2 on day 5). After treatment with 3β-Adiol, a maximum antiproliferative effect was observed on day 6 when cells were cultured in defined serum replacement (reduction of viable cells to 80.4%), whereas cell numbers were decreased to 80.9% on day 4 when cultured in FCS.

### Increased proliferation of OAW-42 cells after knockdown of ERβ

**Fig. 3:**
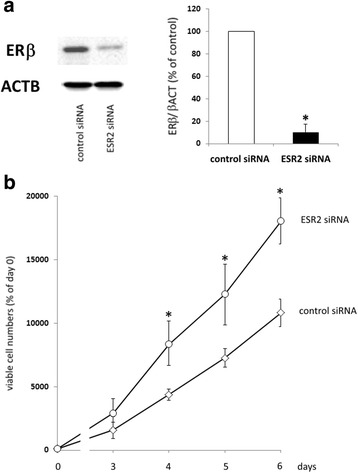
Effect of an ERβ knockdown on proliferation of OAW-42 cells. **a**: ERβ expression in OAW-42 ovarian cancer cells after transfection with ERβ siRNA compared to controls. 72 h after transfection, total protein was isolated and knockdown was examined on the protein level by means of Western blot analysis as described in the methods section. ERβ expression levels after transfection with a mix of ESR2 siRNAs (10 nM each) were compared to levels in cells transfected with negative control siRNA (*n* = 4). *p < 0.01 vs. control-transfected cells. **b**: Proliferation of OAW-42 cells with reduced levels of ERβ. Cells were transfected with ESR2-specific siRNA or negative control siRNA and seeded into 96-well plates (1000 cells/well) in medium containing 10% FCS the next day. 0, 3, 4, 5, and 6 days after transfection, relative numbers of viable cells were determined by means of the fluorimetric CellTiter-Blue^®^ Assay (Promega). From one vial of transfected cells, 72 h after transfection total RNA and protein was isolated in parallel to confirm knockdown of ESR2 expression. Data are expressed in percent of day 0 (*n* = 4). *p < 0.01 vs. control-transfected cells

After having shown a decrease of ovarian cancer cell proliferation resulting from treatment with ERβ agonists, we examined, whether knockdown of ERβ would have the opposite effect. In OAW-42 cells, 72 h after transfection with ESR2 siRNA, Western blot analysis revealed maximum suppression of ERβ protein levels down to 10,5% (*p* < 0.01) (Fig 3a). In OVCAR-3 cells, siRNA treatment resulted in a knockdown of ERβ by 65.7% only, although different transfection parameters were tested (data not shown). Since this knockdown was not sufficient, we had to continue with OAW-42 cells only. When OAW-42 cells were seeded 48 h after siRNA transfection for assessment of proliferation, we observed a significant increased growth rate of cells transfected with ESR2 siRNA compared to negative control siRNA. This effect was present from day 4 until day 6 of the proliferation assay, with a maximum effect of ESR2 siRNA on day 4, resulting in a 1.9-fold increase of viable cells (*p* < 0.01) (Fig. 3b).

### Drug effects on the transcriptome of OVCAR-3 and OAW-42 cells

To analyze the molecular mechanisms underlying the antiproliferative effect of ERβ agonists, we employed Affymetrix Human GeneChips 1.0 to analyze the effect of ERB-041, Liquiritigenin and WAY200070 on transcriptome of both cell lines. While changes of the transcriptome were smaller than expected, cell line OAW-42 was found to be more sensitive to treatment with ERβ agonists in terms of gene expression changes than OVCAR-3 cells. Whereas in OAW-42 cells 3 genes were induced and 9 were downregulated more than 2-fold by at least one of the drugs, in OVCAR-3 cells transcript levels of only 3 genes were found to be decreased more than 2-fold. Among the upregulated genes, *C6ORF99* and *TPTE2* were more than 2-fold increased in OAW-42 cells by two different ERβ agonists (Table 1). In OVCAR-3 cells, expression of the genes *LCN1* and *C21ORF94* was more than 2-fold decreased after treatment with ERB-041 and Liquiritigenin. LCN1 gene was also found to be downregulated by ERB-041 in OAW-42 cells. In the latter line, other significantly downregulated genes were *PTCH2, SNORD25, ND6 and SNORD1.*

**Table 1:** Genes regulated after treatment of the indicated ovarian cancer cell lines with the specific ERβ agonists ERB-041, Liquiritigenin (LIQ.) and WAY − 2,000,070 for 48 h. Shown are genes with at least 2-fold regulation in one experimental setting (values in italics). Data were assessed by means of Affymetrix GeneChip 1.0 microarray analyses and are expressed in -fold change compared to the vehicle control

	OAW-42			OVCAR-3		
	ERB-041	LIQ.	WAY200070	ERB-041	LIQ.	WAY200070
Up-regulated genes						
C6ORF99	*2,52*	*3,81*	1,91	1,35	1,01	-1,17
TPTE2	1,67	*2,05*	*2,26*	1,05	1,22	1,08
CD177	1,55	−1,08	*2,14*	1,53	1,62	1,79
Down-regulated genes						
LINC00314	1,24	-1,26	-1,44	-1,86	*−2,09*	*-2,71*
EPCAM	-1,35	−1,41	*-2,20*	−1,21	-1,02	-1,05
SNORD25	*-2,07*	−1,07	*−2,00*	−1,03	−1,11	-1,07
RNU4-2	-1,46	*−2,09*	−1,49	−1,16	−1,21	-1,03
RNU2-1	-1,62	-1,57	*−2,05*	−1,29	−1,03	-1,30
PTCH2	-1,67	−1,76	*−2,08*	−1,37	−1,10	-1,33
RNU5B-1	-1,51	-1,79	*−2,54*	−1,11	−1,23	-1,09
ND6	*-2,11*	*−2,12*	*−4,01*	−1,38	−1,11	1,42
FAM48B2	-1,29	−1,30	−1,73	*−2,11*	−1,72	-1,76
LCN1	*-2,28*	−1,12	−1,11	*−2.14*	*−2,38*	-1,61
SNORA1	-1,82	*−2,07*	*−2,09*	−1,39	−1,41	−1,71

**Fig. 4:**
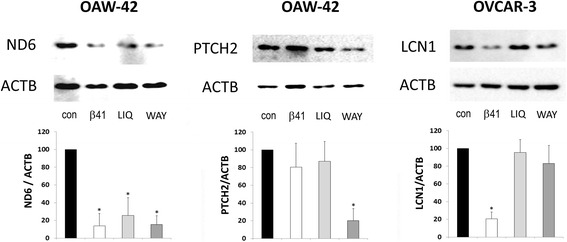
Western blot analysis demonstrating down-regulated protein expression of the indicated genes after treatment with the ERβ agonists ERβ-041, WAY200070 and Liquiritigenin. 72 h after stimulation with 10 nM of the agonists, total protein was isolated and subjected to Western blot analysis. Analyses were performed using specific antibodies against the gene products of LCN1, ND6 and PTCH2 and additionally ACTB as a loading control. Shown are representative results and the densitometrical mean values in relation to ACTB (*n* = 3). *p < 0.01 vs. vehicle

To confirm the results of DNA microarray analysis on the protein level, we performed Western blot experiments to study the effects of ERβ agonists on protein expression of four of those genes most considerably regulated on the mRNA level. In these experiments, we observed strong down-regulation of PTCH2 protein by WAY200070 down to 18.7% in OAW-42 cells (*p* < 0.01), decrease of LCN1 by agonist ERβ-041 down to 21.3% in OVCAR-3 cells (*p* < 0.01). ND6 protein levels in OAW-42 cells decreased down to 13.9% after treatment with ERβ-041 (*p* < 0.01), to 25,5% by Liquiritigenin (*p* < 0.01) and to 15.4% by WAY200070 (*p* < 0.01) (Fig. 4). In contrast, we did not observe a significant effect of the ERβ agonists tested on protein expression of EpCAM which was suggested by microarray results (data not shown).

**Fig. 5:**
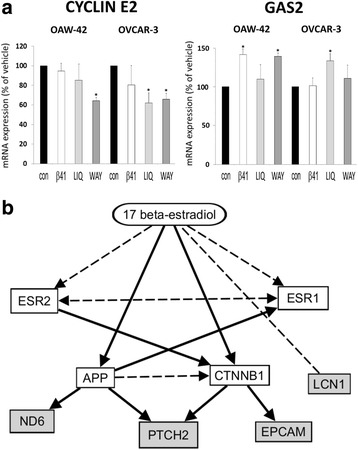
Effect of ERβ agonists on gene expression (Affymetrix GeneChip analysis). **a** Regulation of growth-associated genes *cyclin E2* and *growth arrest specific 2* (GAS2) after treatment with the agonists ERB-041 (β41), Liquiritigenin (LIQ), WAY200070 (WAY) or the vehicle control (con) for 48 h (10 nM) *p < 0.05 bs. Vehicle. **b** Network connecting ESR2 with the genes LCN1, EPCAM, PTCH2 and ND6 being downregulated by ERβ agonists in this study. Broken lines: direct binding. Solid lines: affecting expression. Prediction by IPA Software (Ingenuity Pathway Analysis, Ingenuity Systems, Stanford, USA) [54–61]

DNA Microarray analyses also revealed agonist-triggered regulation of two growth-associated genes which might be an underlying mechanism of the observed growth inhibition. Cyclin E2 (*CCNE2*) expression was found to be decreased after treatment with ERβ agonist Liquiritigenin by 38.6% in OVCAR-3 cells and by 32.8% after treatment with WAY200070 in the same cell line (both *p* < 0.05). In OAW-42 cells, the latter agonist reduced cyclin E2 expression by 35.1% (*p* < 0,05). In contrast, expression of growth arrest specific 2 (*GAS2*) gene was elevated after treatment with ERβ agonists ERB-041 and WAY200070 in OAW-42 cells (by 42.5% or 37.0%, respectively, *p* < 0.05), and in OVCAR-3 cells by 31.6% after treatment with Liquiritigenin (Fig. 5a).

### Pathway analysis

Analysis of the transcriptome changes triggered by ERβ agonists using Ingenuity Pathway Analysis software (IPA, Ingenuity Systems) revealed an estrogen-dependent network consisting of the downregulated genes *LCN1*, *EpCAM*, *PTCH2* and *ND6* (Fig. 5b).

## Discussion

In this study, for the first time we report significant inhibitory effects of ERβ agonists on growth of ovarian cancer cell lines. In turn we demonstrated a significant proliferation increase after siRNA-mediated knockdown of ERβ, corroborating both our agonist findings and the suggested tumor suppressor role of this receptor in ovarian cancer. Though all ERβ agonists inhibited ovarian cancer cell growth, their effect on gene expression partially differed due to their known structural differences.

In ovarian cancer, steroid hormone receptors ERα and β are commonly expressed. Especially in normal ovarian tissue ERβ shows high expression levels, which decrease during carcinogenesis [3, 14, 15, 23–26]. This loss of ERβ could be an important step for the development of ovarian cancer and might even be a general mechanism during tumorigenesis of estrogen-dependent tissues. A number of in vitro studies, including one from our group, support the tumor-suppressive role of ERβ in ovaries [20, 27–33].

The results of our knockdown experiments, clearly suggesting an antiproliferative effect of ERβ in ovarian cancer cells, are in line with previous studies by us and others, reporting growth inhibition after overexpression of ERβ or growth increase after knockdown of this receptor [17, 20].

In our study we addressed the question, whether expression of ERβ in ovarian cancer cells still might be high enough to make this receptor a potential target in ovarian cancer therapy. Thus, we investigated how ovarian cancer cells responded to treatment with ERβ agonists, which have been reported to bind preferentially to this receptor, but only to a much smaller extent to ERα. 3β-Adiol (5α-androstane-3β, 17β-diol) is a dihydrotestosterone metabolite which does not bind androgen receptors. However, it efficiently binds ERβ [34] and acts as a physiological ERβ-activator in different tissues [35, 36]. ERB-041 and WAY-200070 are highly specific synthetic ERβ agonists [37, 38]. ERB-041 is known to display a more than 200-fold selectivity for ERβ than for ERα (EC_50_ ERβ = 2 nM), WAY-200070 still has a 68-fold higher selectivity for ERβ than for ERα (EC_50_ ERβ = 2 nM [39]). Liquiritigenin is a plant-derived flavonoid from licorice root, which acts as a highly selective agonist of ERβ (EC_50_ ERβ = 36.5 nM [40]). Recently, we have shown that Liquiritigenin and 3β-Adiol inhibit proliferation of different breast cancer cell lines. However, proliferation of ERα-positive breast cancer cell lines was not affected by the agonists WAY200070 and ERB-041 [41, 42]. We decided to use a 10 nM concentration of the agonists only, because the EC50 values for ERβ binding of all drugs are in the low nanomolar range, and possible ERβ-unspecific effects of higher drug concentrations on proliferation e.g. via ERα activation thus could be ruled out. Though all agonists affected proliferation regardless of the serum supplement used, our observation that agonist effects in the presence of 10% FCS were higher on OVCAR-3, but lower in OAW-42 cells compared to defined growth-factor free serum replacement might be explained by the different mutation status of these cell lines. OAW-42 cells derive from ascites from a serous ovarian cancer, they obtain mutations of *BRCA1* and *PIK3CA,* but not of *p53* [43]. OVCAR-3 cells were attained from ascites of a patient with high-grade serous ovarian cancer (G3) and exhibit a mutation of *p53* [43]. Thus, proliferation of OVCAR-3 cells, which is elevated due to mutated p53 and is further increased by growth factors, might be more sensitive to growth inhibition by ERβ agonists [44].

The transcriptome analyses of both cell lines we performed after treatment with ERβ agonists ERB-041, Liquiritigenin and WAY-200070 revealed possible molecular mechanisms underlying the observed antiproliferative effects. In our study we observed down-regulation of *PTCH2* in OAW-42 cells both on the mRNA and protein level after treatment with ERβ agonist WAY200070. *PTCH2* gene encodes a transmembrane receptor and is part of the hedgehog signaling pathway, which is known to play an important role in the development of several malignancies [45–49]. High expression of *PTCH2* was associated with a poorer survival in patients with bladder cancer [47]. Recently, Worley et al. showed a significant overexpression of *PTCH2* in ovarian clear cell carcinoma and associated endometriosis [50]. Given that knockdown of PTCH2 was reported to exert significant growth inhibition in a clear cell cancer cell line, this gene might be in part responsible for the observed growth inhibitory effects of this ERβ agonist [50].

Pathway analysis suggested that the observed effects of ERβ agonists are mediated by β-catenin (CTNNB1) and amyloid β precursor protein (APP), which have been reported to form a complex [51]. Expression of *APP* and *CTNNB1* previously has been reported to be inducible by estrogens [52, 53]. *CTNNB1* activity has been reported to be inhibited by *ESR2* and is known to affect expression of *EpCAM* and *PTCH2*, which could explain the link between ERβ agonists and decreased expression of *PTCH2* and *EpCAM* we observed in OAW-42 cells [54–56]. The fact that estrogen-inducible *APP* has been reported to increase expression of *ND6* and *PTCH2* provides a putative molecular mechanism between *ESR2* knockdown and the observed downregulation of *ND6* and *PTCH2* [57, 58].

Our observation of *LCN1* downregulation particularly by ERB-041 in both cell lines could be explained by the fact that E2 has been reported to regulate *LCN1* gene expression [59, 60]. The role of this transporter of small lipophilic ligands in cancer is unclear. However, it remains to be investigated whether *LCN1* might exert tumor-promoting functions like its family member *LCN2* known to induce epithelial to mesenchymal transition and to promote breast cancer invasion in an ERα-dependent manner [61, 62].

## Conclusions

In this study, we were able to demonstrate a significant decrease of proliferation of two ovarian cancer cell lines triggered by different ERβ agonists. Microarray analyses revealed a set of cancer-associated genes being regulated by these agonists. This and the observed increase of proliferation after ERβ knockdown suggest an important role of this receptor in growth control of ovarian cancer cells. Our data suggest, that ERβ could be a promising target for therapy of ovarian cancer. To what extent ERβ agonists could be suitable in the clinical setting has to be examined in further studies.

## Abbreviations

DMEM: Dulbecco’s modified eagle’s medium
DNA: Deoxyribonucleic acid
ERβ: Estrogen receptor beta
FCS: Fetal calf serum
RNA: Ribonucleic acid
siRNA: Short interfering ribonucleic acid
